# Emotional Gaze: The Effects of Gaze Direction on the Perception of Facial Emotions

**DOI:** 10.3389/fpsyg.2021.684357

**Published:** 2021-08-02

**Authors:** Jing Liang, Yu-Qing Zou, Si-Yi Liang, Yu-Wei Wu, Wen-Jing Yan

**Affiliations:** ^1^School of Educational Science, Ludong University, Yantai, China; ^2^Faculty of Psychology, Southwest University, Chongqing, China; ^3^College of Teacher Education, Wenzhou University, Wenzhou, China; ^4^Wenzhou Business College, Wenzhou, China; ^5^School of Mental Health, Wenzhou Medical University, Wenzhou, China

**Keywords:** gaze, emotion perception, facial expression, culture, shared signal theory

## Abstract

Previous research has found that when gaze direction matches the underlying behavioral intent communicated by the expression of a specific emotion, it enhances or facilitates the perception of that emotion; this is called the shared signal hypothesis (SSH). Specifically, a direct gaze shares an approach-orientated signal with the emotions of anger and joy, whereas an averted gaze shares an avoidance-orientated signal with fear and sadness. In this research, we attempted to verify the SSH by using different materials on Asian participants. In Experiment 1 we employed photos of models exhibiting direct and averted gazes for rating tasks, in order to study the effects of gaze direction on participants’ perception of emotion. In Experiment 2 we utilized smiling faces in a similar investigation. The results show that for neutral and smiling faces, a direct gaze (relative to a gaze of avoidance) increased the likelihood of a subject perceiving a happy mood; a gaze of avoidance increased the likelihood that anger and fear would be perceived. The effect of gaze direction on emotional expression perception was verified, but a “facilitating-impairing” pattern was not. The difference between our work and previous research may be attributable to the materials employed (which were more ecological), as well as the participants, who were from a different culture.

## Introduction

Among all nonverbal signals, gaze is one of the most attractive facial features and conveys much information (Admoni and Scassellati, 2017). Perceiving others’ eyes serves distinct social and emotional functions. A gaze usually indicates interest (approach-avoidance), since people often look at things they like and avoid things they don’t (Shimojo et al., 2003; Bayliss et al., 2007). Moreover, gaze can have a significant influence on emotional expression perception. For example, angry people often stare into the eyes of the person with whom they are trying to quarrel or fight, and timid people who fear others may drop their eyes and look away. Previous research has found that when gaze direction matches the underlying behavioral intent (approach-avoidance) communicated by an emotional expression, the perception of that emotion is enhanced or facilitated (Adams and Kleck, 2003, 2005).

These findings can be explained by the shared signal hypothesis (SSH) (Rigato et al., 2013). The SSH proposes that when different cues convey the same information (such as approaching or avoidance), attributions can be made with increased certainty, facilitating the processing of either cue. Interference can occur when different cues convey conflicting information, affecting the processing of each. It has been argued that proximity-oriented emotions such as happiness, love, and anger tend to be expressed through direct vision, while avoidance-oriented emotions such as jealousy, sadness, and disgust are more likely to be communicated by avoidance (Argyle and Cook, 1976; Kleinke, 1986; Fehr and Exline, 1987). For example, angry faces are detected faster and perceived with greater intensity when the expresser’s gaze is directed toward the observer rather than away, while an averted gaze enhances the perception and detection of fearful faces (Rigato et al., 2013). In addition, the SSH may be valid from infancy. Previous work has shown that both adults and infants have demonstrated the ability to discern approach- and avoidance-oriented emotions, matching them with direct and averted gazes, respectively.

In Adams and Kleck (2003) research, it was observed that angry facial expressions were more quickly decoded (i.e., there was less reaction latency in recognition) when displayed in conjunction with a direct rather than averted gaze; in contrast, fear expressions were more quickly decoded when displayed in conjunction with an averted rather than direct gaze. Joyful expressions were more quickly decoded when displayed with a direct rather than averted gaze, and sadness expressions were more quickly decoded when displayed with an averted rather than direct gaze. In Adams and Kleck (2005) work, the SSH was further supported by three studies. The researchers found that a direct gaze led to more angry and joyful disposition attributions, whereas an averted gaze led to more fearful and sad disposition attributions. Benton (2010) examined reactions to briefly presented direct and averted faces displaying expressions of fear and anger; the results supported the notion of signal congruence as a mechanism through which gaze and viewpoint affect our responses to facial expressions. These are very interesting findings and provide a more profound understanding of the SSH and the relationship between gaze direction and emotional expression.

However, the work of Adams and Kleck (2003) faced certain challenges. Bindemann et al. (2008) conducted six experiments to re-examine their results. The latter study indicated that the perception of emotional expression was impaired in a speed-based classification task when the eyes of the face stimulus were averted. In rating tasks, the results were not incongruent, indicating that the perception of selected expressions enhanced under an averted gaze was stimulus was task-bound. Overall, the findings of Adams and Kleck (2003) seemed not to be robust, and instead were situationally dependent. Bindemann et al. (2008) believed it was unresolved how approach/avoidance theory mapped onto existing psychological data, including in previous studies conducted with fearful faces. There is extensive evidence that the perception of fear is intimately linked with a direct eye gaze, characterized by a wide scleral contrast above the iris that disappears as the eyes are turned sideways.

Importantly, the notion that an angry expression involves staring directly at another is not universal, especially taking cultural differences into consideration. Culture has the power to shape not only one’s worldview, but also broad psychological functions such as perception, attention, and cognition (Markus and Kitayama, 1991; Varnum et al., 2010). In many East Asian contexts, direct eye contact can be perceived as threatening and disrespectful, and an averted eye gaze is more appropriate; in many Western contexts the reverse is true. For example, angry averted gazes may be interpreted as an attempt to mitigate the negative emotional arousal of the target and maintain the relationship (Kitayama et al., 2006; Park et al., 2018). Moreover, an averted gaze may show dislike and withdrawal from the intention to attack, and thus is a more suitable way to indicate anger in many situations. From this perspective, facial expressions with an averted gaze are more likely to be perceived as angry in Asian cultures, according to the SSH. Therefore, we are curious about whether such a variety of styles of the SSH for the emotion-gaze relationship also exist for Chinese people, who put a much greater premium on collectivism than individualism (Oyserman et al., 2002).

In addition to reexamining the study by Adams and Kleck (2005), the present research tested the effects of gaze on smiling faces. Smiles are usually considered an approachable facial expression and indication of happiness. However, many psychologists agree that smiles can reflect a vast array of emotions, rather than a simple universal expression of happiness. People may smile when they’re frightened, flirtatious, horrified, or mortified (Ambadar et al., 2009). The emotion associated with a smile is easily affected by gaze direction (e.g., an embarrassed smile reveals itself through an averted gaze). As previous findings on neutral faces have shown, smiling faces may also be more likely to be rated higher for sadness and fear, or such a mixture may generate other emotions.

The manipulation of gaze type (i.e., direct vs. averted) can be accomplished by using an image processing tool such as Photoshop. Such operations provide strict control because only the gaze direction is changed. However, a manually crafted gaze is not particularly natural and sometimes can look strange. As seen in Figure 1 in Ganel (2011), the eyes seemed to be devoid of spirit and may have exerted an unwanted influence over the observers. As Bindemann et al. (2008) claimed, the advantage of “real gaze” stimuli is the reflection of natural variations in eye gaze direction, while the disadvantage is that the stimuli may vary slightly in ways other than gaze, even within the same identity.

In this research, we studied how gaze affects people’s emotional rating of neutral and smiling faces. In Experiment 1 we asked the models to display both direct and averted gazes and investigated whether previous findings held true in different situations. We further investigated smiles with direct and averted gazes in Experiment 2 to explore how the direction of the gaze changed the perception of a smile.

## Experiment 1

### Method

The procedure received ethical approval from the IRB at the university, and written informed consent was obtained from all participants.

**FIGURE 1:**
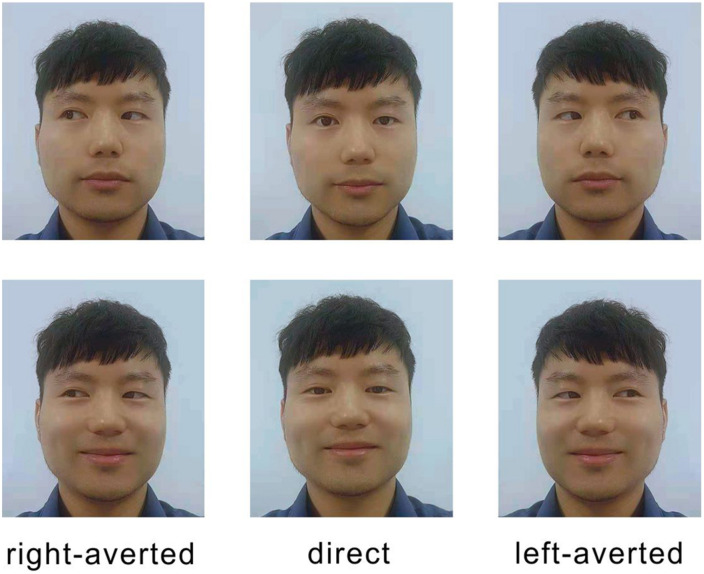
Demo of the materials used in the experiment. The first and second rows indicate the neutral and smiling faces, respectively.

To acquire photos showing averted and direct gazes in both smiling and neutral faces, we recruited 42 college students (21 males and 21 females) and took their pictures; these served as stimuli for the experiment. Participants read and signed consent forms and agreed that their photos could be used for scientific research. Two cameras were mounted on tripods at a height of 120 cm and placed in front of each participant. Individuals were asked to sit on a chair, hold a remote control, look at the middle camera positioned straight ahead of them (with their nose pointed toward the middle camera), and press the remote control to take the photo. We asked participants to display both neutral and smiling faces. To ensure that the participants were smiling naturally, we tried to amuse them by providing recordings of laughter or teasing them. During the entire procedure, the faces were recorded in the full-frontal position. All of the photographs were cropped in order to display only the head and neck of each individual. We copied and mirrored right-gaze photos to obtain left-gaze pictures. In our experiment, each direct-gaze photo was presented twice to balance out the design. A model displaying both left- and right-averted gazes could balance the “direction effect” (if there is any) and generate more stable results for each model in the four trials. All were resized to a standard 320 pixels in width, with variable heights to preserve the aspect ratio (see Figure 1). In this experiment, we used 60 photos from 20 models randomly.

### Participants and Experiment Apparatus

A power analysis by G^∗^Power^1^ 3.1.9.21 indicated *N* = 36 to detect an effect size of 0.25, which is a large effect, with a repeated-measures analysis of variance (ANOVA) and within-factors test (*F*-test), with a number of groups one and number of measurements four, with a probability of 1–β = 0.95, α = 0.05. Assuming possibly invalid or missing data, we recruited more participants than were required. A total of 44 undergraduate students (age: 22.71 ± 1.38 years; 27 females) participated in this study as raters of face photos. All signed consent forms. We used computers with 21-inch LCD monitors (resolution at 1024 × 768 pixels) and employed the software package E-prime 2.0 for stimulus presentation and data collection.

### Design and Procedure

Each participant was seated at a distance of approximately 70 cm from the presentation screen at separate tables in the lab. The researchers verbally explained the participants’ task to make sure they understood how to proceed. All faces showed neutral displays and were presented in random order. Participants were asked to rate each of 80 faces on four emotion scales (i.e., anger, fear, sadness, and joy). There was a 1-min break after 40 trials. The instructions were as follows: “Please rate each of the faces on four emotion scales (anger, fear, sadness, and joy) and enter the associated number in the input box. Ratings are made on a seven-point continuous scale ranging from 1 (not at all frequently) to seven (very frequently).” Each trial began with a fixation cross at the center of the screen that lasted for a duration of 500 ms. This was then replaced by one of the stimulus faces, which remained on the screen until a response was made. Ratings were made on a seven-point continuous scale ranging from 1 (not at all frequently) to seven (very frequently). The next trial began immediately after the preceding response was made.

### Results

**FIGURE 2:**
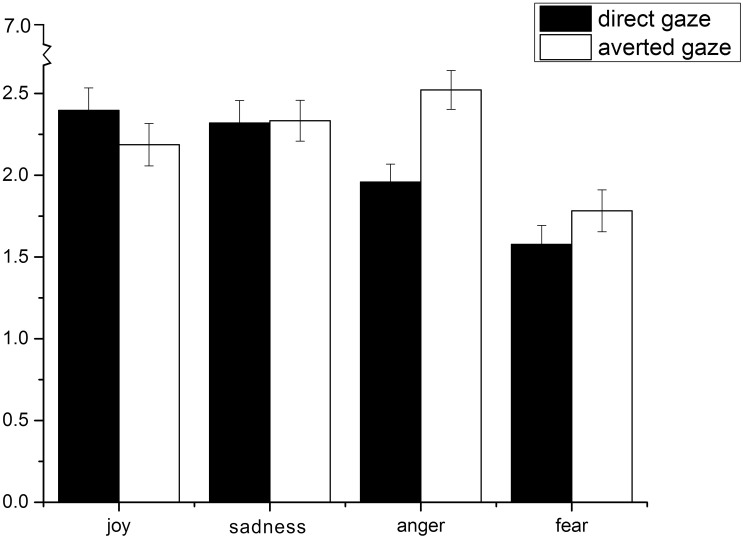
Effects of gaze direction of neutral faces on perceived emotion dispositions. It should be noted that the *Y*-axis indicates a continuous rating scale, which actually ranged from 1 (not at all frequently) to 7 (very frequently) for the task.

In order to test the predicted interaction between gaze direction and perceived emotion disposition, a four (anger/joy/fear/sadness emotion dimension) × 2 (direct/averted gaze direction) repeated measures ANOVA was conducted. A main effect for emotion was found, *F*(3,129) = 11.565, *p* < 0.001, $\eta_{\text{p}}^{2}$ = 0.212, indicating that the emotion rating was different between types, regardless of gaze direction (see Figure 2). The post hoc test showed that faces were rated lower for the fear disposition than for other emotion dispositions (fear = 1.68, anger = 2.24, sadness = 2.33, joy = 2.29). A main effect also emerged for gaze, *F*(1,43) = 32.428, *p* < 0.001, $\eta_{\text{p}}^{2}$ = 0.430, such that faces showing an averted gaze were rated higher overall for the likelihood to experience emotion (*M* = 2.21, SE = 0.091) than were faces showing a direct gaze (*M* = 2.06, SE = 0.101).

Consistent with our expectations, an emotion-gaze interaction was found, *F*(3,129) = 40.315, *p* < 0.001, $\eta_{\text{p}}^{2}$ = 0.484, indicating that the direction of the gaze resulted in different effects on perceptions of emotion. A simple effects analysis was conducted to assess these effects for each emotion condition. As predicted, a direct gaze (*M* = 2.40, SE = 0.92), relative to an averted gaze (*M* = 2.19, SE = 0.87), increased the perceived likelihood of the stimulus person being perceived as having a joyful disposition, *t*(43) = 6.125, *p* < 0.01, *r* = 0.969. Conversely, an averted gaze (*M* = 2.51, SE = 0.80), relative to a direct gaze (*M* = 1.94, SE = 0.72), was more often associated with an angry disposition, *t*(43) = −8.953, *p* < 0.001, *r* = 0.851. Likewise, an averted gaze (*M* = 1.72, SE = 0.76), relative to a direct gaze (*M* = 1.51, SE = 0.62), was more often associated with a fearful disposition, *t*(43) = −3.707, *p* = 0.001, *r* = 0.901.

### Discussion

This study found that gaze direction systematically influenced the perceived emotion disposition conveyed by a neutral face. In this experiment, a direct gaze was attributed to a more joyful disposition, whereas an averted gaze was attributed to an angrier or more fearful disposition. However, Adams and Kleck found that a direct gaze enhanced the perception of approach (i.e., anger and joy) and improved the mood of approach (i.e., anger and joy), while avoiding eye contact enhanced the perception of avoidance (i.e., fear and sadness). In this experiment, as compared to an averted gaze, a direct gaze increased the subject’s perception of a happy mood; an averted gaze increased the perception of anger and fear relative to a direct gaze. The differences in our findings could be due to variations in the materials used in the experiment, as well as cultural incongruities. These will be discussed in the general discussion below.

The next step was to study whether gaze direction would affect the perception of emotion in smiling expressions. Smiling is an approaching signal, but when it is accompanied by an averted gaze, it is more likely to be judged as sadness or fear, as previous findings on neutral faces have found. Alternatively, the sense of anger and fear might be blocked by the smile. In Experiment 2 we changed the intensity of the facial expressions, using a smiling expression to determine whether doing so would affect the role of gaze direction in emotion perception.

## Experiment 2

In the pre-experiment, we randomly presented neutral and smiling faces to participants. However, the results showed that the neutral and smiling faces influenced one another. The smiling faces exhibited strong emotions, which made the neutral faces overwhelmingly more likely to be rated as neutral. Therefore, in Experiment 2 we only presented smiling faces.

### Method

A total of 28 female and nine male undergraduate students participated in this study. Individuals were asked to rate the facial displays regarding the emotion expressed, using an emotion profile comprized of a number of scales (i.e., happiness, anger, fear, and sadness) ranging from 1 (not at all frequently) to 7 (very frequently). Except for all faces displaying positive emotions, all other conditions were the same as in Experiment 1.

### Results

**FIGURE 3:**
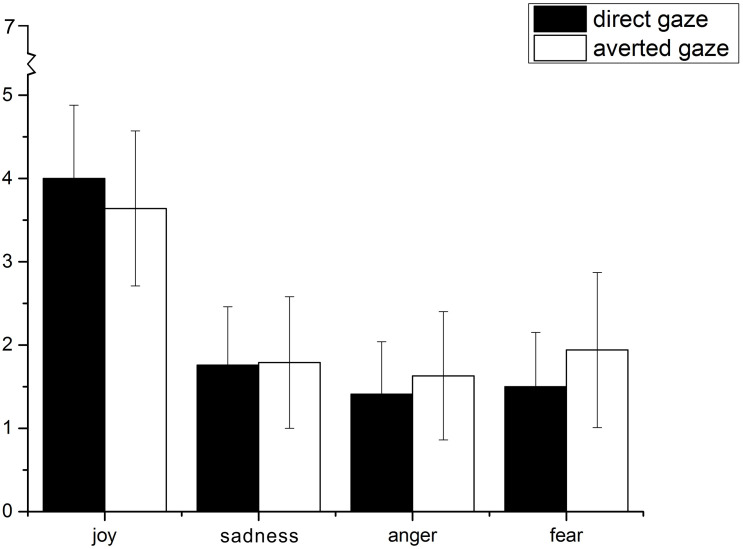
Effects of gaze direction of smiling faces on perceived emotions. It should be noted that the Y-axis indicates a continuous rating scale that ranged from 1 (not at all frequently) to 7 (very frequently) for the task.

In order to test the predicted interaction between gaze direction and perceived emotion disposition, a 4 (anger/joy/fear/sadness emotion dimension) × 2 (direct/averted gaze direction) repeated measures ANOVA was computed. A main effect for emotion was found, *F*(3,108) = 132.016, *p* < 0.001, $\eta_{\text{p}}^{2}$ = 0.786. Inspection of the means indicated that this effect was due to the faces being rated higher with regards to the joyful disposition than the other emotion dispositions (joy = 3.80, sadness = 1.77, anger = 1.52, fear = 1.72). A main effect also emerged for gaze, *F*(1,36) = 8.614, *p* < 0.05, $\eta_{\text{p}}^{2}$ = 0.193, such that averted gaze faces were rated higher overall with regards to the likelihood of experiencing emotion (*M* = 2.25, SE = 0.101) than were direct gaze faces (*M* = 2.19, SE = 0.089) (see Figure 3). Consistent with our expectations, these main effects were qualified by an emotion/gaze direction interaction, *F*(3,108) = 16.388, *p* < 0.001, $\eta_{\text{p}}^{2}$ = 0.313.

Direct *t*-tests were then conducted to assess the reliability of these effects for each emotion condition. As predicted, the direct gaze (*M* = 4.00, SE = 0.88), relative to the averted gaze (*M* = 3.64, SE = 0.93), increased the likelihood of the stimulus person being perceived as having a joyful disposition, *t*(36) = 4.26, *p* < 0.001. Conversely, an averted gaze (*M* = 1.63, SE = 0.77), relative to a direct gaze (*M* = 1.41, SE = 0.63), was more often associated with an angry disposition, *t*(36) = −3.132, *p* < 0.05. Likewise, an averted gaze (*M* = 1.94, SE = 0.93), relative to a direct gaze (*M* = 1.50, SE = 0.65), was more often associated with a fearful disposition, *t*(36) = −4.588, *p* < 0.001.

## General Discussion

This study found that gaze direction affected people’s perceptions of emotion in neutral expressions, but the pattern identified was different from that uncovered in previous research. Adams and Kleck (2005) found that the direction of the eyes’ gaze affected the emotions perceived, depending on the specific type of emotion; a direct gaze could improve the mood of approach (i.e., anger and joy), while a gaze avoiding direct eye contact could enhance the perception of avoidance (i.e., fear and sadness). In Experiment 1 a direct gaze, relative to a gaze of avoidance, increased the likelihood of a subject perceiving a happy mood; a gaze of avoidance increased the likelihood that anger and fear would be perceived. Neutral faces had no emotional tendency, and thus were easily affected by gaze direction. However, when we changed the emotion type in Experiment 2 by using smiling faces, we also found that the perception of emotion (usually positive emotions) could be significantly modulated by gaze direction within the same pattern. Eye gaze could modulate the perception of emotion not only in neutral faces, but also for other types of facial expressions.

However, in our study, the “facilitating-impairing” pattern displayed was different from that found in Adams and Kleck (2005). One reason for this may be that we used different materials and designs. The materials used in Adams and Kleck (2005) is different in several aspects: (1) half the faces were manipulated to display direct gaze and half averted gaze; (2) gaze direction was manipulated using Adobe Photoshop; (3) if an exemplar face was presented with direct gaze in one stimulus set, it was presented with averted gaze in the other set. In our study, the “within-stimulus” approach was chosen to better control for individual differences because we considered that the approach-avoidance feeling is largely affected by the model’s appearance. Moreover, in the present study, gaze direction was directly provided by participants. This operation was the same as what was used in Bindemann et al. (2008), but their results showed that real and manually crafted gazes produced similar results. Averting the eyes may also affect other facial muscles and somewhat distort general facial expressions. For example, when participants tried to avert their eyes without adjusting their head orientation, they felt unnatural and not as comfortable as usual. Our pattern was also different from what was found in Bindemann et al. (2008), where an averted gaze impaired overall emotional expression perception. They used both real and manually crafted gazes and obtained similar results. In addition, compared with the design in Adams and Kleck (2005), the participants in the present study were more likely to remember their earlier ratings, consequently increasing the similarity of the ratings for direct and averted gazes. The results show that even though there might have been such an impact, the effect size was still large. This design proves that the effect of gaze direction is very robust from another point of view.

In addition, cultural differences may have been responsible for this incongruity. Researchers have noted that individualism and independent cultural expressions emphasize the direct and explicit communication of emotions (Markus and Kitayama, 1991), while in collectivist cultures such as those of East Asia, the suppression of emotions is more encouraged than is emotional expression (Soto et al., 2011). In fact, in Western cultures, people tend to practice independent self-interpretation; denying oneself emotional expression and experience is equivalent to denying one’s true self. Conversely, in Asian countries such as Japan, China, and South Korea, people are more collectivist and interdependent. Controlling and suppressing emotional expressions is considered key to maintaining a harmonious relationship with the group (Markus and Kitayama, 1991); direct expressions of emotion have a negative impact on interpersonal and collective relationships. Anger and an averted gaze seem to be culturally congruent in these countries. This is partly supported by Park et al. (2018), where P1 in the Angry-Averted condition was significantly larger than P1 in the Neutral-Averted condition (*p* < 0.005). The conclusion was that Asian Americans allocated more early attentional processing to averted angry eyes, as compared to neutral and direct angry gazes. The authors explained that averted gazes may reflect a culturally appropriate emotional attunement and could be interpreted as an attempt to mitigate the negative emotional arousal of the target and an attempt to maintain the relationship. Therefore, in Eastern cultures, people tend to avert their eyes to reduce the negative impact of interpersonal communication; thus, averted eyes are more likely to be perceived as angry. From this perspective, the SSH is also supported by our research, though the pattern may be different due to the different “shared signal pattern.” In the current study, a direct gaze shared an approach-orientated signal with joy, whereas an averted gaze shared an avoidance-orientated signal with fear and anger.

There are some limitations in the present study. We used only neutral and smile faces as the materials. It is likely that the spontaneity of the smiles could be a potential factor that affect the emotion perception and the elicited smiles in the present study may vary in spontaneity. In addition, in the future studies different facial expression should be further considered. As for the manually crafted gaze, it should be compared with the real directly in the future study and the results could be more persuasive when the differences were explained by cultural differences.

This study used ecological experiment materials to study the effects of eye orientation on the perception of emotion by Chinese subjects. The SSH and effect of gaze direction on emotional expression perception were verified for neural and smiling faces, but the SSH pattern could be affected by various factors. Whether such differences are due to the materials, procedure, or culture is a topic requiring further investigation.

## Data Availability Statement

The raw data supporting the conclusions of this article will be made available by the authors, without undue reservation.

## Ethics Statement

The studies involving human participants were reviewed and approved by IRB in Wenzhou University. The patients/participants provided their written informed consent to participate in this study.

## Author Contributions

W-JY and JL conceived and designed the experiments. Y-WW and Y-QZ performed the experiments. Y-QZ, JL, S-YL, and W-JY analyzed the data. JL, Y-QZ, S-YL, and W-JY wrote and revised the manuscript.

## Conflict of Interest

The authors declare that the research was conducted in the absence of any commercial or financial relationships that could be construed as a potential conflict of interest.

## Publisher’s Note

All claims expressed in this article are solely those of the authors and do not necessarily represent those of their affiliated organizations, or those of the publisher, the editors and the reviewers. Any product that may be evaluated in this article, or claim that may be made by its manufacturer, is not guaranteed or endorsed by the publisher.

## Footnotes

1 G^∗^Power is a tool used to compute statistical power analyses. G^∗^Power can also be used to compute effect sizes and graphically display the results of power analyses. http://stats.idre.ucla.edu/other/gpower/.
